# On potential ocular artefacts in infant electroencephalogram: a reply to comments by Köster

**DOI:** 10.1098/rspb.2016.1285

**Published:** 2016-07-27

**Authors:** Dora Kampis, Eugenio Parise, Gergely Csibra, Ágnes Melinda Kovács

**Affiliations:** 1 Department of Cognitive Science, Central European University, Budapest 1015, Hungary; 2 Department of Psychology, Flyde College, Lancaster University, Lancaster LA1 4YF, UK; 3 Department of Psychological Sciences, Birkbeck, University of London, London WC1E 7HX, UK

Köster's comment [1] on Kampis *et al.* [2] adopts an objection that was put forward previously regarding the interpretation of scalp-recorded gamma-band electroencephalogram (EEG) activity in adults as a correlate of object processing. Gamma-band (over 25 Hz) oscillatory activity has been consistently found to signal object processing in various populations, such as non-human primates, human adults and human infants. However, Yuval-Greenberg *et al.* [3] reported that in human adults saccadic spike potentials (SPs), co-occurring with microsaccades (MSs), contribute to this signal, and questioned the neural origins of the oscillatory activation found in earlier studies. In response to this, specific tools have been developed to remove possible MS-related artefacts from adult EEG data (e.g. Hassler *et al.* [4]).

Köster [1] points out that analogous attempts have not been implemented in infancy research. We argue that while this is indeed the case, there are several theoretical and methodological considerations that cast doubt on whether it is necessary or possible to apply these tools to infant EEG recordings.

First, the algorithm applied on adult EEG to remove MS-related artefacts would not be applicable to infant recordings as it is. Hassler *et al.* [4] propose a two-step method, which consists of detecting and then removing SPs that accompany MSs. The first step of this method detects SPs based on their characteristics in adult EEG. However, Csibra *et al.* [5] found no saccade-related SPs in infants younger than 12 months, and even at this age SPs differed greatly in amplitude and in morphology from those reported in adults. Because of this, the algorithms used with adults to detect SPs would simply not be applicable to infant EEG. The second step of Hassler *et al.* [4], using independent component analysis (ICA) to remove MS-related SPs from the signal, also seems unfeasible to apply directly on infant data given the nature of infant EEG recordings. As Köster [1] rightly points out, performing ICA requires a vast amount of data to produce valid results. As an estimate, finding *N* stable components in *N*-channel data requires more than 3 × *N*^2^ sample points at each channel [6]. In EEG recordings at 128 channels and 500 Hz sampling rate (as in our study) this requirement demands more than 90 s of perfectly clean EEG on *all* channels. In most infant EEG studies (especially ones with relatively longer trials and dynamic stimuli), recordings are regularly contaminated by movement artefacts, and the cleaned data are much sparser than what might be required by ICA.

Furthermore, to our knowledge no one has managed to identify and measure MSs in infants so far, and therefore it is not known in what form they occur at this early age. While the appropriate tools are available (eye-trackers with a high enough sampling rate), it would be a separate methodological challenge to keep a young infant's head sufficiently stable for accurately measuring MSs. Therefore, even in case of successful co-recording of EEG and eye movements, it is unclear how MSs (and/or SPs) should be detected. Because of this, at the moment it is not possible to remove any potential MS-related artefacts from infant EEG, and we agree with Köster [1] that we cannot decisively exclude the possibility that MS contaminate gamma-band responses in infants.

To estimate the likelihood of eye movement contamination of our measures in Kampis *et al.* [2], we performed an additional analysis on our time–frequency data from Study 1. To approximate a measure of eye movement-related activity, we estimated the bipolar horizontal EOG signal in our recordings by subtracting the activation at the two electrodes closest to the outer canthi of the eyes (channels 1 and 32) from each other. We then subjected this signal to the same time–frequency analysis as our original data and correlated the resulted gamma activation in this EOG signal with the activation we obtained in our original analyses. If eye movements induced the gamma-band activation found in our study, then activations at the temporal channels would probably be correlated with the EOG signal. However, this correlation was not significant either in segment 1 (*r* = 0.347, *p* = 0.205 in occlusion condition—for activations see figure 1; and *r* = 0.239, *p* = 0.390 in control condition) or in segment 2 (*r* = −0.059, *p* = 0.835 in occlusion condition and *r* = −0.099, *p* = 0.725 in control condition). Based on this analysis, it seems unlikely that our findings originate from eye movements.

**Figure 1.**
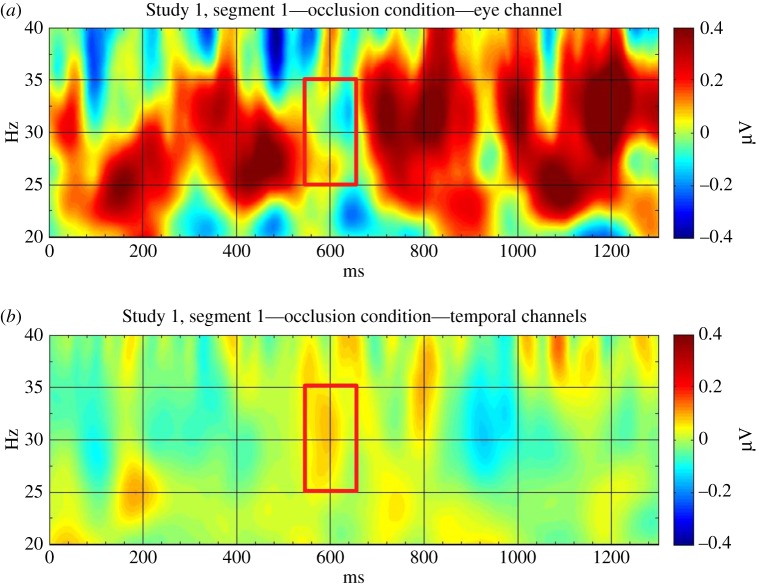
Gamma-band activation in (*a*) the eye channel (channel 32 subtracted from channel 1) and (*b*) temporal channels (channels 40, 41, 46, 47, 51, 97, 98, 102, 103, 109). The red rectangles mark the frequency and time window used in the analyses in Kampis *et al.* [2]. (Online version in colour.)

Additionally, beyond the methodological challenge to detect MS-related artefacts in infant EEG, several findings (including some mentioned by Köster [1]) of scalp-recorded gamma-band activity during object processing in infants would not be easily explained by MS patterns. First, in many cases there were no visual differences during the measurement periods between the experimental and control conditions, and therefore it is not clear why MSs would show a different pattern (e.g. [7,8]). Second, many of the studies reported gamma-band activity over temporal areas (e.g. [2,7]), whereas MS-related SPs were found mostly around the midline in adults [3]. Third, while MS-related SPs were shown to manifest themselves in a time window of approximately 200–350 ms after stimulus onset, many studies have used different time windows for analyses (e.g. [2,9]), and in some cases it is not obvious what should count as stimulus onset, as activation was measured after a longer sequence of events [2,7]. Finally, as Melloni *et al.* [10] pointed out in their response to the paper demonstrating MS-related gamma activity, MS-related EEG effects should show a broadband response, whereas many studies report effects in narrower gamma ranges, and this observation also applies to infant recordings.

In sum, on the one hand the tools developed for MS-related artefact removal from adult EEG are not used currently in infant EEG because they are not straightforwardly applicable to infant data. Once our understanding of the characteristics of infant EEG and (oculo-)motor development reaches the necessary level, it will be possible to return to these concerns and address them.

On the other hand, it is not clear whether this issue has to be addressed in infants, as the factors that were found to induce possible artefacts in adult studies are not simply hard to measure but might not be present (or might have radically different characteristics) in young infants. With regard to our own data [2], it seems unlikely that the gamma-band activation in temporal areas was due to infants' eye movements during the observation of the events (figure 1). Finally, some recent results, also discussed by Köster [1], suggest that gamma-band oscillations, even in the adult literature, provide us with a valid tool to investigate object representations [11].

## Data accessibility

The EEG dataset used in the analyses reported in this article are available at: http://dx.doi.org/10.5061/dryad.k1r85.
